# Patient‐Derived iPSC‐MSC Modelling Reveals Reversible GSK3‐Mediated Suppression of Mesenchymal Lineage Differentiation in Legg‐Calvé‐Perthes Disease

**DOI:** 10.1111/jcmm.71350

**Published:** 2026-09-07

**Authors:** Binna Seol, Hyung Jin Lim, Cho Lok Song, Jungwoon Lee, Yee Sook Cho

**Affiliations:** ^1^ Stem Cell Research Laboratory, Immunotherapy Research Center Korea Research Institute of Bioscience and Biotechnology (KRIBB) Daejeon South Korea; ^2^ Department of Bioscience, KRIBB School University of Science & Technology Daejeon South Korea; ^3^ Stem Cell & Regenerative Biology Laboratory, Environmental Disease Research Center Korea Research Institute of Bioscience and Biotechnology (KRIBB) Daejeon South Korea

**Keywords:** GSK‐3β, iPSC, Legg‐Calvé‐Perthes disease, mesenchymal stem cell, Wnt signalling

## Abstract

Legg‐Calvé‐Perthes disease (LCPD) is an idiopathic paediatric osteonecrosis characterized by avascular injury to the femoral head, resulting in structural collapse and early osteoarthritis. Although mesenchymal stem cell (MSC) dysfunction has been implicated, the underlying mechanisms remain unclear due to the absence of human disease models. Here, we generated patient‐specific induced pluripotent stem cells (iPSCs) from dermal fibroblasts of LCPD patients and differentiated them into mesenchymal stem cells (iMSCs) to establish an in vitro platform for mechanistic interrogation. LCPD‐iMSCs exhibited hallmark disease‐associated phenotypes, including concurrent suppression of osteogenic, adipogenic and chondrogenic differentiation, accompanied by enhanced phosphorylation of GSK3α/β, reduced β‐catenin abundance and attenuated canonical Wnt signalling. Transcriptomic analyses revealed alterations in extracellular matrix organization, adhesion pathways and PI3K‐Akt/MAPK signalling, consistent with a cellular environment predisposed to impaired mesenchymal function. Pharmacological inhibition of GSK3 using lithium chloride restored β‐catenin levels, improved osteogenic and adipogenic marker expression, and enhanced matrix deposition, demonstrating functional reversibility of the LCPD phenotype. These findings identify GSK3‐mediated β‐catenin destabilization as a central regulator of MSC lineage impairment in LCPD and establish a patient‐derived iPSC‐MSC model as a valuable platform for mechanistic and therapeutic exploration in paediatric osteonecrosis.

## 1 Introduction

Legg‐Calvé‐Perthes disease (LCPD) is an idiopathic avascular osteonecrosis of the femoral head in children and is classified within the broader spectrum of osteonecrosis of the femoral head (ONFH) [1, 2]. The disease is initiated by transient interruption of the blood supply to the capital femoral epiphysis, resulting in ischemic necrosis of osteocytes and bone marrow cells and progressive weakening of the subchondral trabecular bone. Although the overlying articular cartilage initially remains viable because it receives nutrients from synovial fluid, continued mechanical loading on the weakened epiphysis ultimately leads to femoral head deformation, loss of sphericity, secondary cartilage degeneration and early‐onset osteoarthritis [3, 4]. Each year, an estimated 20,000–30,000 new osteonecrosis cases are diagnosed in the United States, representing approximately 10% of all hip arthroplasties [5]. Despite this clinical burden, particularly among children, LCPD pathogenesis remains poorly understood, and current treatments remain largely supportive rather than regenerative. The immature femoral head is especially susceptible to deformation following ischemic injury, and management strategies vary from conservative containment to surgical reconstruction, with many patients eventually requiring joint replacement [6]. Although radiography and magnetic resonance imaging (MRI) improve diagnostic accuracy [7], no available therapy directly addresses the cellular dysfunction underlying disease progression.

Animal models have greatly advanced the understanding of LCPD. Porcine and rodent models have reproduced key radiological and histopathological features of human disease, demonstrating that ischemia induces upregulation of hypoxia‐inducible factor 1α (HIF‐1α), which interacts with Sox9 to influence chondrogenesis [8]. Ischemic activation of HIF‐1α also contributes to inflammation, including increased IL‐6 levels and synovitis [9]. Elevated IL‐6 and HMGB1 in synovial fluid from children with active LCPD suggest that inflammatory signalling participates in disease progression [10, 11, 12]. Recent studies further indicate that LCPD is not solely a consequence of vascular insufficiency but also involves complex molecular and cellular responses, including oxidative stress, abnormal angiogenesis, extracellular matrix remodelling and dysregulated signalling pathways that collectively impair skeletal repair and femoral head remodelling. Nevertheless, the intrinsic cellular mechanisms linking these pathological processes to defective skeletal regeneration remain incompletely understood.

Mesenchymal stromal/stem cells (MSCs) play essential roles in skeletal homeostasis and repair through their capacity for osteogenic, adipogenic and chondrogenic differentiation. As the principal progenitor cells responsible for bone, cartilage and stromal tissue regeneration, coordinated multilineage differentiation is fundamental to normal MSC function. In ONFH and related osteonecrotic conditions, MSCs have been reported to exhibit reduced proliferative and osteogenic capacity [13, 14, 15, 16]. Canonical Wnt/β‐catenin signalling is a key regulator of MSC differentiation, promoting osteogenesis and chondrogenesis while suppressing adipogenesis through β‐catenin stabilization and activation of downstream transcriptional programs [17, 18, 19]. Conversely, hyperactivation of glycogen synthase kinase‐3β (GSK‐3β), a central negative regulator of β‐catenin, disrupts Wnt signalling and has been implicated in impaired osteogenic differentiation in osteonecrosis models. Pharmacological targeting of the Wnt/GSK‐3β axis has shown promise. Lithium chloride (LiCl), a GSK‐3β inhibitor, enhances osteogenesis by stabilizing β‐catenin and inducing osteogenic genes, and has demonstrated therapeutic effects in both rodent ONFH models and patient‐derived MSCs [13, 14, 20]. However, whether LCPD is associated with intrinsic defects in MSC lineage commitment remains unknown, largely because of the limited availability of appropriate human disease models capable of separating cell‐autonomous abnormalities from secondary effects of the ischemic tissue environment. Furthermore, translational application of bone marrow‐derived MSCs is limited by their scarcity and impaired regenerative potential in osteonecrotic bone [21, 22, 23, 24, 25, 26].

Induced pluripotent stem cells (iPSCs) overcome these limitations by providing a renewable, patient‐specific source of MSCs (iMSCs). Disease modelling using iPSC‐MSCs has successfully recapitulated pathological phenotypes in ONFH and demonstrated their utility for evaluating targeted therapies [27]. Because patient‐derived iMSCs can be generated and differentiated under standardized conditions, they provide a unique opportunity to evaluate intrinsic mesenchymal phenotypes while minimizing confounding influences from the diseased tissue microenvironment. In this study, we generated patient‐specific iPSCs from children with LCPD and differentiated them into MSCs to establish a human in vitro disease model. LCPD‐derived iMSCs exhibited concurrent suppression of osteogenic, adipogenic and chondrogenic differentiation, together with hyperactivation of GSK3 signalling and reduced β‐catenin availability, indicating a broad impairment in mesenchymal lineage competency. We further demonstrate that pharmacological GSK3 inhibition restores β‐catenin levels and rescues multilineage differentiation capacity. Collectively, these findings identify reversible dysregulation of the GSK3/β‐catenin axis as a key mechanism underlying intrinsic MSC dysfunction in LCPD and establish a patient‐specific iPSC‐MSC platform for mechanistic studies and the development of regenerative therapeutic strategies for paediatric osteonecrosis.

## 2 Materials and Methods

### 2.1 Primary Cells

Human dermal fibroblasts (CRL‐2097) were obtained from the American Type Culture Collection (ATCC, Manassas, VA, USA). Two Legg‐Calvé‐Perthes disease (LCPD) fibroblast lines, ASE‐5055 and AI001‐F‐LCP, were purchased from Applied StemCell Inc. (Milpitas, CA, USA) and DV Biologics (Yorba Linda, CA, USA), respectively. Cells were cultured in α‐minimum essential medium (α‐MEM; Thermo Fisher Scientific, Waltham, MA, USA) supplemented with 15% fetal bovine serum (FBS; Thermo Fisher Scientific), 1% penicillin–streptomycin (P/S; Thermo Fisher Scientific), 1× non‐essential amino acids (NEAA; Thermo Fisher Scientific), and 1% sodium pyruvate (Thermo Fisher Scientific). Cultures were maintained at 37°C in a humidified atmosphere containing 5% CO_2_. Cells were used for experiments between passages 4 and 7. Mycoplasma contamination was routinely monitored using the MycoAlert Mycoplasma Detection Kit (Lonza, Basel, Switzerland).

### 2.2 Generation and Culture of Human Induced Pluripotent Stem Cells (hiPSCs)

Integration‐free wild‐type and LCPD patient‐derived hiPSCs were generated using the CytoTune‐iPS 2.0 Sendai Reprogramming Kit (Thermo Fisher Scientific, A16517), which delivers *OCT3/4, SOX2*, *KLF4* and *c‐MYC* (OSKM) via non‐integrating Sendai viral vectors [28]. On Day 0, 1 × 10^5^ fibroblasts were seeded per well in six‐well culture plates (Corning Inc., Corning, NY, USA). On Day 1, cells were transduced with Sendai viral vectors at multiplicity of infection (MOI) ratios of 5:5:3 for hKOS (human *KLF4*, *OCT3/4* and *SOX2*), hc‐*MYC*, and h*KLF4*, respectively. On Day 5 post‐transduction, cells were harvested and replated onto γ‐irradiated mouse embryonic fibroblasts (MEFs; 1–5 × 10^4^ cells/well; GlobalStem, Gaithersburg, MD, USA) and maintained in hiPSC medium consisting of 80% DMEM/F12 (Thermo Fisher Scientific), 20% KnockOut Serum Replacement (KSR; Thermo Fisher Scientific), 1× NEAA (Thermo Fisher Scientific), 1× GlutaMAX I (Thermo Fisher Scientific), and 0.1 mM β‐mercaptoethanol (Sigma‐Aldrich, St. Louis, MO, USA), supplemented with 8 ng/mL basic fibroblast growth factor (FGF2; PeproTech, Rocky Hill, NJ, USA). The medium was replaced every other day.

Emerging colonies with embryonic stem cell (ESC)‐like morphology were manually selected between Days 14–21 and expanded using 0.5 mM EDTA in Dulbecco's phosphate‐buffered saline (DPBS; Thermo Fisher Scientific). Cultures were passaged until Sendai virus clearance was confirmed by RT‐PCR using the CytoTune 2.0 Detection Kit (Thermo Fisher Scientific). Two patient‐specific hiPSC lines, LCPD‐iPSC‐1 (derived from ASE‐5055) and LCPD‐iPSC‐2 (derived from AI001), were established and used for subsequent experiments. All iPSC lines were maintained between passages 10 and 20 for downstream analyses.

### 2.3 Alkaline Phosphatase Assay

hiPSCs were fixed in 4% paraformaldehyde (PFA; Sigma‐Aldrich, St. Louis, MO, USA) for 2 min and stained using the Alkaline Phosphatase Detection Kit (Sigma‐Aldrich). Staining was visualized using an Axio Observer inverted microscope (Carl Zeiss, Oberkochen, Germany).

### 2.4 Immunocytochemistry

Cells were fixed in 4% PFA for 10 min, permeabilized with 0.1% Triton X‐100 in DPBS for 30 min, and blocked with 4% bovine serum albumin (BSA; Sigma‐Aldrich) for 1 h at room temperature (RT). Primary antibodies against OCT4, NANOG, TRA‐1‐60, TRA‐1‐81, SSEA‐3, SSEA‐4 and β‐catenin were incubated overnight at 4°C at a 1:200 dilution. Alexa Fluor 488‐ or 594‐conjugated secondary antibodies (Thermo Fisher Scientific) were applied at a 1:500 dilution for 1 h at RT. Nuclei were counterstained with 1 μg/mL 4′,6‐diamidino‐2‐phenylindole (DAPI; Thermo Fisher Scientific). Images were captured using an Olympus IX81 fluorescence microscope (Olympus, Tokyo, Japan) with identical exposure settings. Negative controls without primary antibody and isotype controls were included to confirm specificity. Quantitative fluorescence analysis was performed using ImageJ software (National Institutes of Health, Bethesda, MD, USA). The antibodies used are listed in Table S1.

### 2.5 Teratoma Assay

Approximately 1–2 × 10^6^ hiPSCs were suspended in growth factor‐reduced Matrigel (1:1 in DPBS) and injected subcutaneously into the dorsal flanks of immunodeficient CAnN.Cg‐*Foxn1* ^nu^ mice (Orient Bio Inc., Seongnam, Korea). After 10 weeks, teratomas were excised, fixed in 4% PFA, paraffin‐embedded, sectioned and stained with haematoxylin and eosin (H&E; Sigma‐Aldrich). A certified veterinary pathologist confirmed the presence of tissues representing all three germ layers. All animal experiments were conducted in compliance with the Institutional Animal Care and Use Committee (IACUC) guidelines of the Korea Research Institute of Bioscience and Biotechnology (KRIBB; approval no. KRIBB‐AEC‐19153).

### 2.6 Differentiation of hiPSCs Into Mesenchymal Stem Cells (iMSCs)

hiPSCs were differentiated into iMSCs using the STEMdiff Mesenchymal Progenitor Kit (STEMCELL Technologies, Vancouver, Canada; #05240) under xeno‐free conditions. Feeder‐free hiPSCs at 80%–90% confluence were induced toward the mesodermal lineage for 4 days and subsequently matured into iMSCs for 17 days on fibronectin‐coated plates (Sigma‐Aldrich). On day 21, the differentiated cells were washed with phosphate‐buffered saline and dissociated using 0.25% trypsin–EDTA (Gibco, Thermo Fisher Scientific, Waltham, MA, USA). The cells were subsequently expanded in MSC growth medium, and iMSCs at passages 3–5 were used for all subsequent experiments.

### 2.7 Flow Cytometric Analysis of iMSC Surface Markers

Detached iMSCs were fixed with 4% PFA for 10 min at RT and stained with fluorophore‐conjugated antibodies against CD34, CD45, CD73, CD90 and CD105 (Miltenyi Biotec, Bergisch Gladbach, Germany). Corresponding isotype controls were included to verify staining specificity. Data acquisition was performed using a BD Accuri C6 flow cytometer (BD Biosciences, San Jose, CA, USA). Dead cells were excluded by DAPI staining. Flow cytometric data were analysed using FlowJo software v10 (BD Biosciences), with gating based on forward‐ and side‐scatter (FSC/SSC) parameters from unstained controls and fluorescence compensation performed using BD CompBeads (BD Biosciences). The list of antibodies used is provided in Table S1.

### 2.8 Cell Growth Analysis

Healthy donor, LCPD‐1 and LCPD‐2 iMSCs were seeded at 3 × 10^4^ cells per well in 12‐well plates, in triplicate for each time point. Cells were cultured for 6 days, and separate wells were harvested on days 0, 1, 2, 3, 4, 5 and 6. At each time point, cells were detached with 0.25% trypsin–EDTA, resuspended in culture medium, and mixed 1:1 with trypan blue solution. Viable, dye‐excluding cells were counted using a haemocytometer. Cell growth curves were generated from the mean viable cell number at each time point from three independent experiments.

### 2.9 Trilineage Differentiation and Histochemical Staining

For lineage differentiation, iMSCs were seeded at 5 × 10^4^ cells/cm^2^ for osteogenic and chondrogenic induction for 21 days and for adipogenic induction for 14 days. Differentiation media were refreshed every 2–3 days. Calcium deposition during osteogenic differentiation was visualized using 2% Alizarin Red S (Sigma‐Aldrich) for 10 min at RT, followed by three washes with distilled water. Lipid droplet accumulation in adipogenic cultures was detected using 05% Oil Red O (SigAldrich) for 10 min at RT, followed by three washes with distilled water. Chondrogenic differentiation was confirmed by staining sulfated glycosaminoglycans with 1% Alcian Blue (Sigma‐Aldrich) in 0.1 M HCl for 30 min at RT, followed by three washes with DPBS. All stained samples were imaged using an (Olympus IX81 inverted microscope) and band intensities were quantified using ImageJ software. Each experiment was performed in triplicate.

### 2.10 Quantitative Real‐Time Reverse Transcription PCR

Total RNA was extracted using the RNeasy Mini Kit (QIAGEN, Hilden, Germany), and 1 μg of RNA was reverse‐transcribed using the SuperScript II First‐Strand Synthesis System (Thermo Fisher Scientific). Quantitative PCR was performed with SYBR Green Master Mix (Applied Biosystems, Foster City, CA, USA) on a 7500 Fast Real‐Time PCR System (Applied Biosystems). The PCR conditions were as follows: 95°C for 10 min, followed by 40 cycles of 95°C for 15 s and 60°C for 60 s, with a subsequent melt‐curve analysis. Gene expression levels were normalized to *GAPDH* using the ΔΔ*C*_t_ method. No‐template controls were included in each run. The primer sequences used are listed in Table S2.

### 2.11 Western Blot Analysis

Protein samples were prepared using RIPA lysis buffer (Sigma‐Aldrich) supplemented with protease and phosphatase inhibitor cocktails (Thermo Fisher Scientific). Protein concentrations were determined using the Pierce BCA Protein Assay Kit (Thermo Fisher Scientific). Equal amounts of protein (20 μg per sample) were separated by 4%–10% SDS‐polyacrylamide gel electrophoresis (SDS‐PAGE) and transferred onto polyvinylidene difluoride (PVDF) membranes (Millipore, Burlington, MA, USA). Membranes were blocked with 5% non‐fat milk in phosphate‐buffered saline containing 0.1% Tween‐20 (PBST) for 1 h at RT, followed by overnight incubation at 4°C with primary antibodies against glycogen synthase kinase‐3β (GSK3β), phospho‐GSK3α/β (Tyr279/Tyr216), β‐catenin, NF‐κB, phospho‐NF‐κB (Ser536), IκBα, and β‐actin, which served as a loading control. After washing, membranes were incubated with horseradish peroxidase (HRP)‐conjugated secondary antibodies and visualized using enhanced chemiluminescence (ECL; Thermo Fisher Scientific). Immunoreactive bands were captured using an Amersham Imager 600 (Cytiva, Marlborough, MA, USA), and band intensities were quantified using ImageJ software. Antibodies used in this study are listed in Table S1.

### 2.12 RNA‐Seq Analysis

Total RNA was isolated from healthy donor fibroblasts (CRL‐2097), LCPD‐1 fibroblasts, LCPD‐2 fibroblasts, healthy donor‐iMSCs, and LCPD‐2‐iMSCs using the RNeasy Mini Kit (QIAGEN) according to the manufacturer's instructions. RNA integrity was assessed using an Agilent 2100 Bioanalyzer with an RNA 6000 Nano Chip (Agilent Technologies, Santa Clara, CA, USA), and RNA concentration was measured using an ND‐2000 spectrophotometer (Thermo Fisher Scientific). RNA sequencing libraries were prepared using the SMARTer Stranded RNA‐Seq Kit (Clontech, Mountain View, CA, USA) and sequenced on an Illumina HiSeq 2500 platform (Illumina, San Diego, CA, USA) to generate 100 bp paired‐end reads. Reads with an average quality score below Q20 were removed during quality filtering. The resulting clean reads were aligned to the human reference genome (UCSC hg19) using the corresponding UCSC transcript annotation to identify known transcripts and potential novel splice junctions. Gene expression levels were normalized by quantile normalization of read counts (RCs). Differential gene expression was calculated as log_2_ fold change relative to the healthy donor control based on normalized read counts. All sequencing and bioinformatic analyses were performed by Ebiogen Inc. (Seoul, Korea). The raw RNA‐seq data generated in this study have been deposited in the NCBI Gene Expression Omnibus (GEO) under accession number *GSE342619*. Processed gene expression data, including normalized expression values and log_2_ fold‐change values, are provided in Tables S3 and S4, respectively.

### 2.13 GSK‐3β Inhibitor Treatment

For inhibition of GSK3β, cells were treated with 5 mM lithium chloride (LiCl; Sigma‐Aldrich) or 2.5 μM CHIR99021 (PeproTech, Cranbury, NJ, USA). CHIR99021 was dissolved in 0.02% dimethyl sulfoxide (DMSO; Sigma‐Aldrich), and control cultures received an equivalent volume of vehicle.

### 2.14 Statistical Analysis

All data are expressed as the mean ± standard deviation (SD) from at least three independent biological replicates. Statistical analyses were performed using unpaired, two‐tailed Student's *t*‐tests. Statistical significance was defined as *p value < 0.05 and **p value < 0.01.

## 3 Results

### 3.1 Generation and Characterization of iPSCs Derived From LCPD Patient Fibroblasts

**FIGURE 1:**
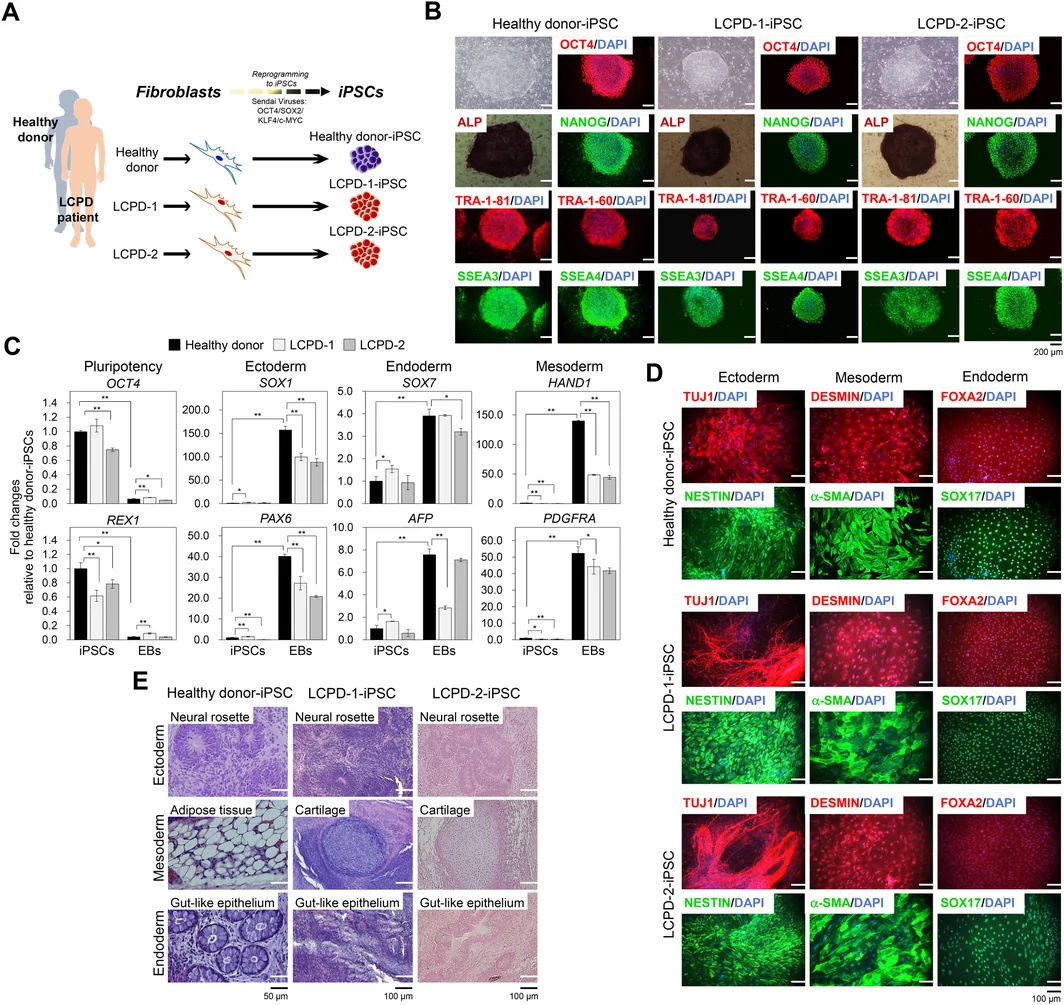
Generation and pluripotency characterization of iPSCs derived from healthy donor and LCPD patient fibroblasts. (A) Schematic illustration of the Sendai virus‐mediated reprogramming of dermal fibroblasts from a healthy donor and two LCPD patients (LCPD‐1 and LCPD‐2) using non‐integrating vectors encoding *OCT4*, *SOX2*, *KLF4* and *c‐MYC*. (B) Pluripotency characterization of established iPSC lines. Representative phase‐contrast and alkaline phosphatase (ALP) staining images of healthy donor‐, LCPD‐1‐ and LCPD‐2‐iPSCs, together with immunofluorescence staining for pluripotency markers OCT4, NANOG, TRA‐1‐60, TRA‐1‐81, SSEA3 and SSEA4. Nuclei were counterstained with DAPI. Scale bar = 200 μm. (C) qRT‐PCR analysis of pluripotency markers *OCT4* and *REX1* in undifferentiated iPSCs and representative ectodermal markers *SOX1* and *PAX6*, mesodermal markers *HAND1* and *PDGFRA*, and endodermal markers *SOX7* and *AFP* following embryoid body‐mediated spontaneous differentiation. (D) In vitro trilineage differentiation potential of healthy donor‐ and LCPD‐iPSCs confirmed by immunostaining for markers of the three germ layers: Ectoderm (TUJ1, NESTIN), mesoderm (DESMIN, α‐SMA) and endoderm (FOXA2, SOX17). Scale bar = 100 μm. (E) In vivo pluripotency validation through teratoma formation in immunodeficient mice. Haematoxylin‐ and eosin‐stained sections show derivatives of ectoderm (neural rosettes), mesoderm (adipose tissue and cartilage) and endoderm (gut‐like epithelium). Scale bar = 50 μm (control) and 100 μm (LCPD‐iPSCs).

To establish a patient‐specific in vitro model of Legg‐Calvé‐Perthes disease (LCPD), dermal fibroblasts from affected patients and a healthy control individual were reprogrammed into induced pluripotent stem cells (iPSCs) using a non‐integrative Sendai virus system expressing *OCT4*, *SOX2*, *KLF4* and *c‐MYC* (Figure 1A). Reprogramming produced compact, embryonic stem cell (ESC)‐like colonies with high nucleus‐to‐cytoplasm ratios and well‐defined colony borders in both patient‐derived lines and the normal control line. These colonies were expanded to establish three stable iPSC lines: healthy donor iPSC, LCPD‐1‐iPSC and LCPD‐2‐iPSC. Standard G‐band karyotyping verified chromosomal stability, revealing normal diploid karyotypes without detectable abnormalities in all three iPSC lines (Figure S1).

All three iPSC lines exhibited strong alkaline phosphatase (ALP) activity, indicating successful reprogramming and acquisition of an undifferentiated pluripotent state (Figure 1B). Immunofluorescence analysis further demonstrated robust expression of the pluripotency‐associated markers OCT4, NANOG, TRA‐1‐60, TRA‐1‐81, SSEA‐3 and SSEA‐4, with comparable staining patterns across healthy donor‐ and LCPD‐derived iPSCs (Figure 1B). To further assess pluripotency and multilineage differentiation potential, qRT‐PCR analysis was performed for representative pluripotency‐ and germ‐layer specific markers in undifferentiated iPSCs and embryoid bodies (EBs) generated by spontaneous differentiation (Figure 1C). Undifferentiated healthy donor‐, LCPD‐1 and LCPD‐2‐iPSCs expressed the pluripotency‐associated genes *OCT4* and *REX1*. Following EB‐mediated spontaneous differentiation, all three lines showed induction of ectodermal markers *SOX1* and *PAX6*, mesodermal markers *HAND1* and *PDGFRA*, and endodermal markers *SOX7* and *AFP*, although the magnitude of induction varied among the individual lines (Figure 1C). These transcriptional changes demonstrated activation of lineage‐specific gene expression programs representing all three embryonic germ layers in both healthy donor‐ and LCPD‐derived iPSCs. Consistent with the qRT‐PCR findings, immunofluorescence analysis of differentiated EBs demonstrated expression of representative lineage‐specific proteins from all three germ layers, including TUJ1 and NESTIN for ectoderm, DESMIN and α‐SMA for mesoderm, and FOXA2 and SOX17 for endoderm (Figure 1D). Thus, the induction of germ layer‐specific transcripts was accompanied by expression of corresponding lineage‐associated proteins, providing complementary evidence of multilineage differentiation capacity in vitro. Pluripotency was further assessed in vivo using teratoma formation assays in immunodeficient mice. Histological analysis revealed structures corresponding to all three germ layers in all iPSC lines, including neural rosettes (ectoderm), adipose tissue and cartilage (mesoderm) and gut‐like epithelium (endoderm) (Figure 1E). Collectively, these findings demonstrate that healthy donor‐ and LCPD‐derived fibroblasts were successfully reprogrammed into iPSCs that maintained normal karyotypes, expressed characteristic pluripotency markers, and retained the capacity for differentiation into derivatives of all three embryonic germ layers in vitro and in vivo. These characterized iPSC lines were subsequently used to generate iMSCs for further investigation of LCPD‐associated cellular phenotypes.

### 3.2 Differentiation of LCPD‐iPSCs Into iMSCs and Surface Marker Characterization

**FIGURE 2:**
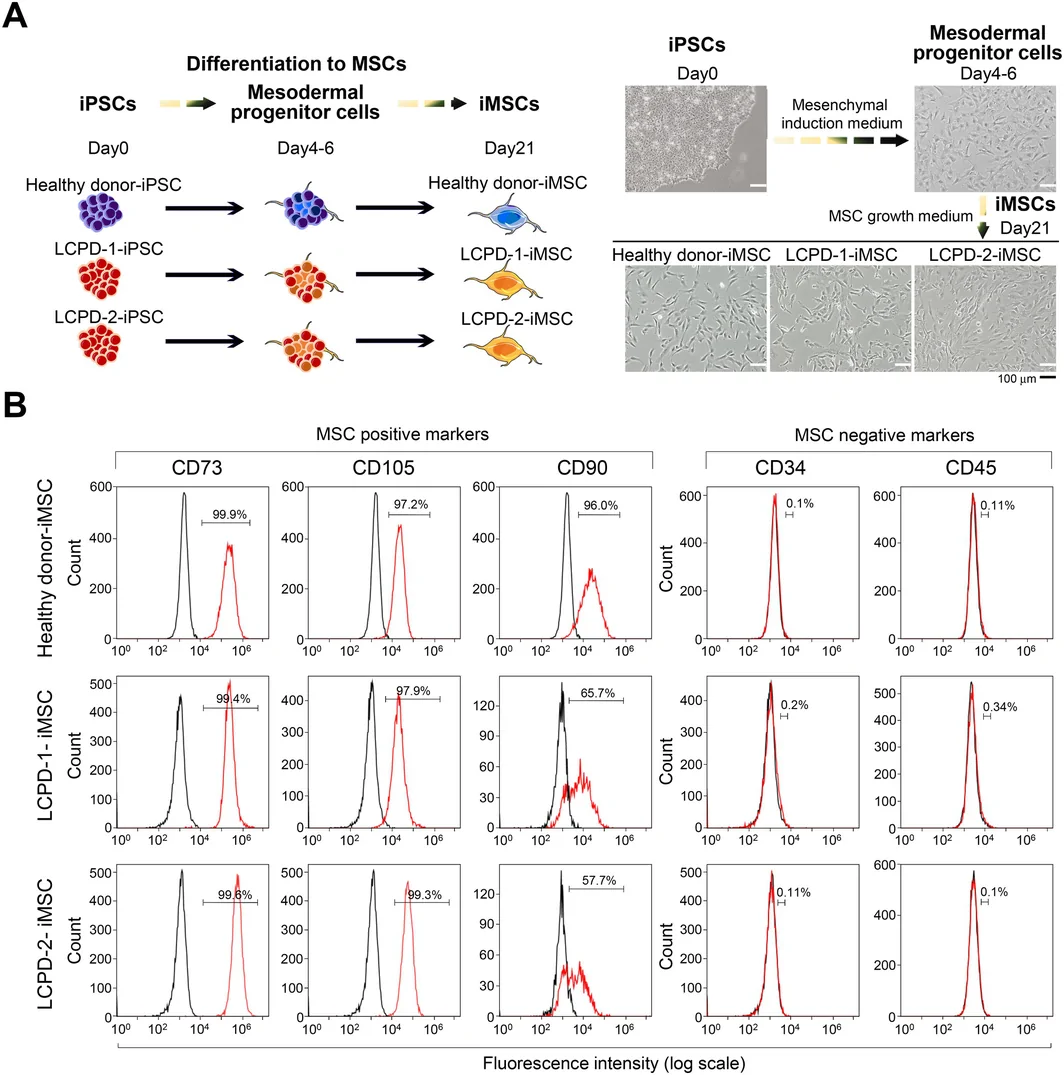
Differentiation of healthy donor‐ and LCPD‐iPSCs into mesenchymal stem cells (iMSCs). (A) Schematic illustration of the stepwise differentiation process from iPSCs to mesodermal progenitor cells (day 4–6) and subsequently to iMSCs (day 21). Representative phase‐contrast images show fibroblast‐like, spindle‐shaped morphology typical of healthy donor‐, LCPD‐1‐ and LCPD‐2‐iMSCs. Scale bar = 100 μm. (B) Flow cytometric analysis of surface marker expression to confirm the MSC phenotype of iMSCs. iMSCs expressed canonical MSC surface markers CD73, CD90 and CD105, while lacking expression of the haematopoietic markers CD34 and CD45. Representative histograms from three independent experiments are shown. The x‐axis represents fluorescence intensity (log scale), and the y‐axis represents the number of recorded events (Count).

To examine whether LCPD‐iPSCs maintain their capacity to generate mesenchymal stem cells (iMSCs), healthy donor‐, LCPD‐1‐ and LCPD‐2‐iPSC lines were subjected to a defined stepwise MSC induction protocol (Figure 2A). After 21 days of differentiation, all groups yielded adherent, spindle‐shaped cells consistent with typical MSC morphology. No noticeable morphological differences were observed among the three iMSC populations. Flow cytometric profiling confirmed successful mesenchymal specification in all lines (Figure 2B). Healthy donor‐iMSCs showed uniformly high expression of canonical MSC markers CD73, CD90 and CD105, and were negative for the haematopoietic markers CD34 and CD45, aligning with established MSC identity criteria (Figure 2B). LCPD‐1‐ and LCPD‐2‐iMSCs similarly expressed CD73 and CD105 at levels comparable to controls, indicating preserved acquisition of these core MSC markers. However, both LCPD‐derived iMSC lines displayed notably reduced CD90 expression (approximately 60%–70%) compared with healthy donor‐iMSCs (approximately 95%). The reduction in CD90, a surface glycoprotein involved in adhesion and MSC immunophenotype, suggests a subtle deviation in phenotypic maturation specific to LCPD‐derived cells. Despite this difference, all iMSC lines remained negative for CD34 and CD45, confirming the absence of haematopoietic contamination. Growth analysis further revealed no significant differences in proliferation among healthy donor‐ and LCPD‐iMSCs (Figure S2), indicating that reduced CD90 expression does not impair cellular expansion. Collectively, these results confirm that LCPD‐iPSCs retain the ability to generate MSC‐like cells but exhibit a subtle phenotypic deviation (lower CD90) that warrants further evaluation in downstream functional assays.

### 3.3 LCPD‐iMSCs Show Reduced Osteogenic, Adipogenic, and Chondrogenic Differentiation Potential

**FIGURE 3:**
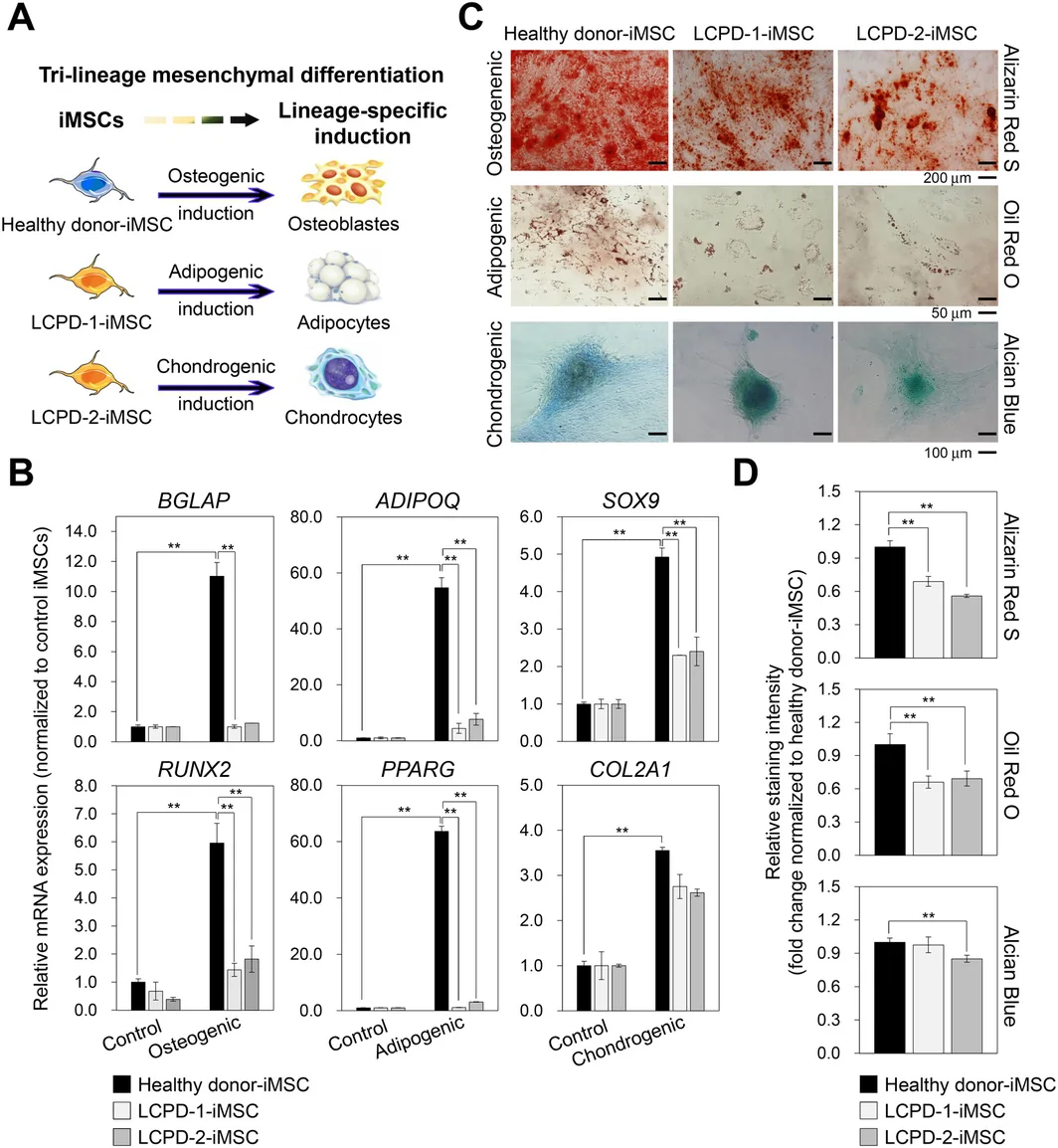
Trilineage differentiation capacity of healthy donor‐ and LCPD‐iMSCs. (A) Schematic illustration of trilineage differentiation from iMSCs into osteogenic, adipogenic and chondrogenic lineages. (B) Quantitative RT‐PCR analysis of lineage‐specific marker genes following differentiation: Osteogenic (*RUNX2*, *BGLAP*), adipogenic (*PPARG*, *ADIPOQ*) and chondrogenic (*SOX9*, *COL2A1*). Data are normalized to *GAPDH* and expressed relative to undifferentiated iMSCs. *p < 0.05 and **p < 0.01 versus healthy donor‐iMSC; *n* = 3 independent experiments. (C) Representative histochemical staining of differentiated iMSCs: Alizarin Red S for calcium deposition (osteogenesis), Oil Red O for lipid droplets (adipogenesis), and Alcian Blue for glycosaminoglycan‐rich matrix (chondrogenesis). Scale bars = 200 μm (Alizarin Red S), 50 μm (Oil Red O) and 100 μm (Alcian Blue). (D) Quantification of staining intensity corresponding to (C). Data are presented as mean ± SD from three independent experiments.

To evaluate the mesenchymal differentiation capacity of LCPD‐derived iMSCs, tri‐lineage differentiation assays were performed under osteogenic, adipogenic and chondrogenic induction conditions (Figure 3A). Healthy donor‐iMSCs efficiently differentiated into all three mesenchymal lineages. In contrast, both LCPD‐1‐iMSCs and LCPD‐2‐iMSCs exhibited broadly impaired differentiation responses, although the extent of inhibition differed among lineages. Under osteogenic conditions, healthy donor‐iMSCs showed robust mineralized matrix formation, as indicated by strong Alizarin Red S staining (Figure 3C). Mineral deposition was markedly reduced in both LCPD‐derived iMSC lines, with quantitative analysis confirming a significant decrease in staining intensity compared with healthy donor‐iMSCs (Figure 3D). Consistently, osteogenic marker genes, including *BGLAP* and *RUNX2*, were strongly induced in healthy donor‐iMSCs but only weakly upregulated in LCPD‐iMSCs following osteogenic induction (Figure 3B), indicating a pronounced defect in osteoblast differentiation. Adipogenic differentiation was similarly compromised in LCPD‐iMSCs. Healthy donor‐iMSCs accumulated abundant lipid droplets upon adipogenic induction, as demonstrated by intense Oil Red O staining, whereas lipid accumulation was substantially reduced in both LCPD‐derived lines (Figure 3C). Quantification confirmed significantly lower adipogenic staining intensity in LCPD‐iMSCs (Figure 3D). At the transcriptional level, the adipogenic regulators *ADIPOQ* and *PPARG* were robustly induced in healthy donor‐iMSCs but showed markedly attenuated expression in LCPD‐iMSCs (Figure 3B), supporting impaired adipocyte differentiation. In contrast, chondrogenic differentiation, although reduced, was relatively better preserved in LCPD‐derived iMSCs compared with osteogenic and adipogenic lineages. Following chondrogenic induction, healthy donor‐iMSCs formed cartilage‐like aggregates with strong Alcian blue staining, reflecting efficient proteoglycan deposition (Figure 3C). Both LCPD‐iMSC lines displayed decreased Alcian blue staining intensity relative to healthy donor‐iMSCs; however, the reduction was less pronounced than that observed for osteogenic and adipogenic differentiation (Figure 3D). Consistently, chondrogenic marker genes *SOX9* and *COL2A1* were induced in LCPD‐iMSCs, albeit at significantly lower levels than in healthy donor‐iMSCs (Figure 3B), indicating partial but insufficient chondrogenic differentiation. Taken together, these findings demonstrate that LCPD‐derived iMSCs exhibit a global impairment in mesenchymal differentiation capacity, with osteogenic and adipogenic lineages being more severely affected, while chondrogenic differentiation remains partially preserved. This pattern suggests that LCPD‐associated defects do not uniformly abolish mesenchymal lineage commitment but instead differentially disrupt lineage‐specific differentiation programs.

### 3.4 LCPD‐iMSCs Exhibit a Coordinated Suppression of β‐Catenin and Hyperactivation of GSK3 Signalling

**FIGURE 4:**
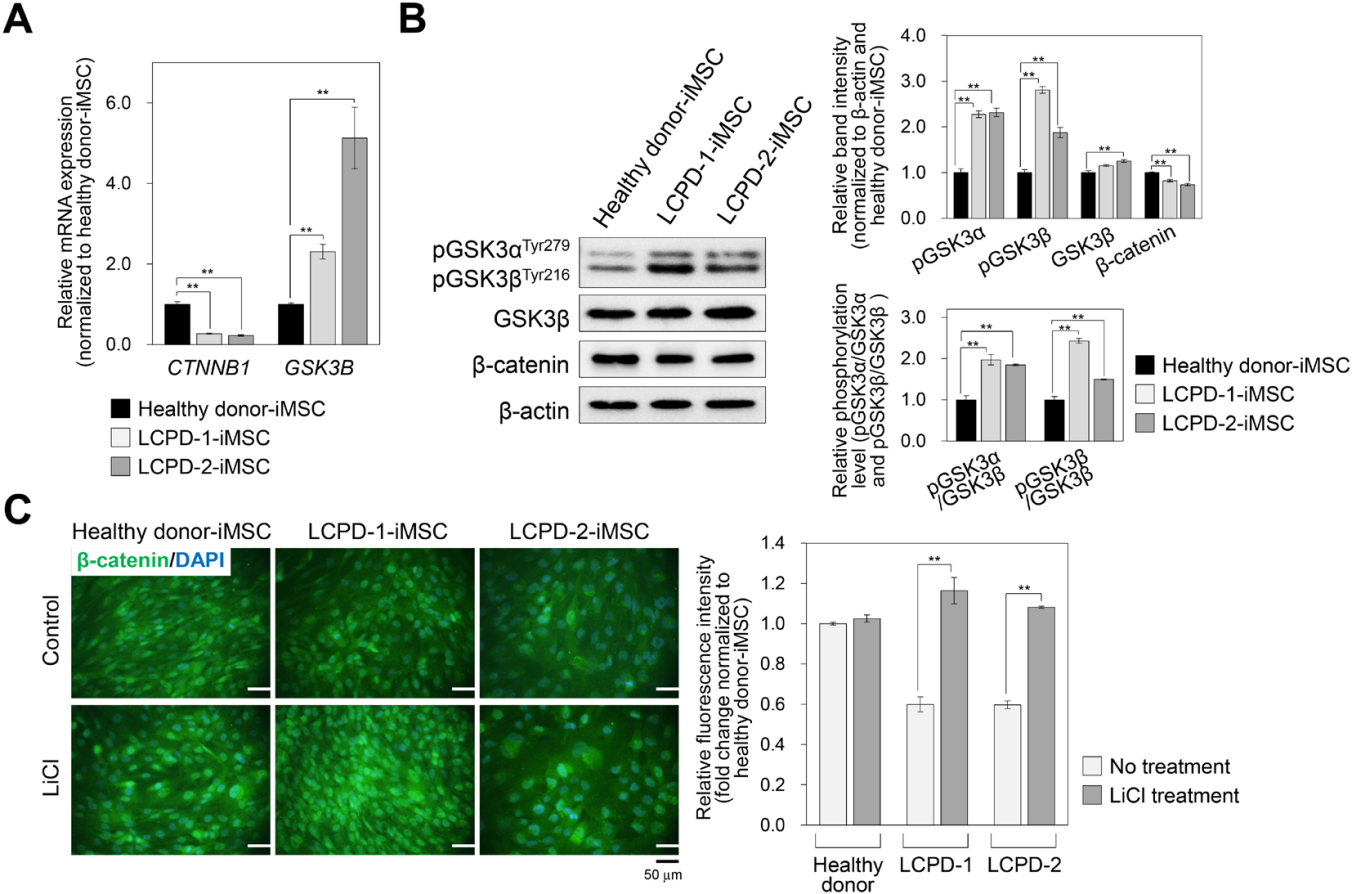
Dysregulation of Wnt/β‐catenin signalling in LCPD‐iMSCs. (A) Quantitative RT‐PCR analysis of CTNNB1 and GSK3B expression in healthy donor‐ and LCPD‐derived iMSCs. Expression levels were normalized to GAPDH. Data represent the mean ± SD from three independent experiments. *p < 0.05 and **p < 0.01 versus healthy donor‐iMSCs. (B) Western blot analysis of total GSK3β, phosphorylated forms (pGSK3α^Tyr279^, pGSK3β^Tyr216^), and β‐catenin in healthy donor‐, LCPD‐1‐ and LCPD‐2‐iMSCs. β‐Actin was used as a loading control. Representative blots (left) and corresponding densitometric quantification (right) are shown. Data are presented as mean ± SD from three independent experiments. (C) Immunofluorescence staining of β‐catenin in untreated (Control) and LiCl‐treated iMSCs. Fluorescence intensity was quantified using ImageJ. Representative images (left) and quantitative analysis (right) are shown. Scale bar = 50 μm. Data represent mean ± SD from three independent experiments. *p < 0.05 and **p < 0.01 versus healthy donor‐iMSCs.

Because impaired osteogenic differentiation in MSCs is tightly regulated by canonical Wnt/β‐catenin signalling, and LCPD‐iMSCs showed reduced osteogenesis in Figure 3, we next investigated whether Wnt pathway components were altered in LCPD‐derived iMSCs. Expression analysis revealed a significant reduction in *CTNNB1* (β‐catenin) transcripts in both LCPD‐1‐ and LCPD‐2‐iMSCs compared with healthy donor‐iMSC, indicating diminished β‐catenin gene expression at baseline (Figure 4A). In contrast, *GSK3B* mRNA was markedly increased in LCPD‐iMSCs, suggesting transcriptional upregulation of a key negative regulator of β‐catenin stability. To determine whether these transcriptional alterations translated into dysregulated protein activity, we examined GSK3α and GSK3β phosphorylation (Figure 4B). Both pGSK3α^Tyr279^ and pGSK3β^Tyr216^, phosphorylation sites associated with enhanced enzymatic activity, were substantially elevated in LCPD‐iMSCs relative to healthy donor‐iMSCs. Quantitative activation ratios (pGSK3α/GSK3β and pGSK3β/GSK3β) further revealed isoform‐ and patient‐specific differences: LCPD‐1‐iMSCs displayed the strongest activation, with pGSK3α and pGSK3β reaching ~1.8–2.2‐fold above healthy donor‐iMSC levels, indicating robust dual‐isoform hyperactivation. LCPD‐2‐iMSCs exhibited similarly increased pGSK3α, but a more modest increase in pGSK3β (~1.4–1.6‐fold), suggesting a relatively greater contribution of GSK3α in this line. Despite these differences, both lines shared the common phenotype of markedly increased GSK3α/β activation relative to total GSK3β, reflecting a disease‐associated shift toward an overall hyperactive GSK3 signalling state. This pattern is consistent with the elevated GSK3B transcript levels detected in LCPD‐derived iMSCs.

Consistent with enhanced GSK3 activity, total β‐catenin protein levels were significantly reduced in both LCPD‐iMSCs. Because GSK3 directly phosphorylates β‐catenin to promote its proteasomal degradation, these results strongly suggest that increased GSK3α/β activation contributes to accelerated β‐catenin turnover in LCPD‐iMSCs. To assess whether β‐catenin reduction also occurred at the cellular level, we performed immunofluorescence staining (Figure 4C). Under control conditions, β‐catenin fluorescence intensity was visibly lower in both patient‐derived lines than in healthy donor‐iMSCs, confirming reduced intracellular β‐catenin abundance. Treatment with lithium chloride (LiCl), a pharmacologic inhibitor of GSK3, increased β‐catenin levels in all lines; however, the magnitude of recovery was greater in LCPD‐iMSCs, restoring β‐catenin fluorescence to levels comparable to untreated healthy donor‐iMSCs. These findings indicate that the β‐catenin deficiency in LCPD‐iMSCs is not an irreversible defect, but rather reflects a reversible, kinase‐dependent regulatory imbalance that can be rescued through GSK3 inhibition. Together, the transcriptional, biochemical, and imaging analyses demonstrate that LCPD‐derived iMSCs exhibit a coordinated pattern of reduced β‐catenin expression and enhanced GSK3α/β activation, providing a mechanistic explanation for the impaired osteogenesis and heightened pathway vulnerability.

### 3.5 LCPD‐iMSCs Show Heightened Inflammatory Responsiveness and Further Suppression of Wnt/β‐Catenin Signalling Upon IL‐1β Stimulation

**FIGURE 5:**
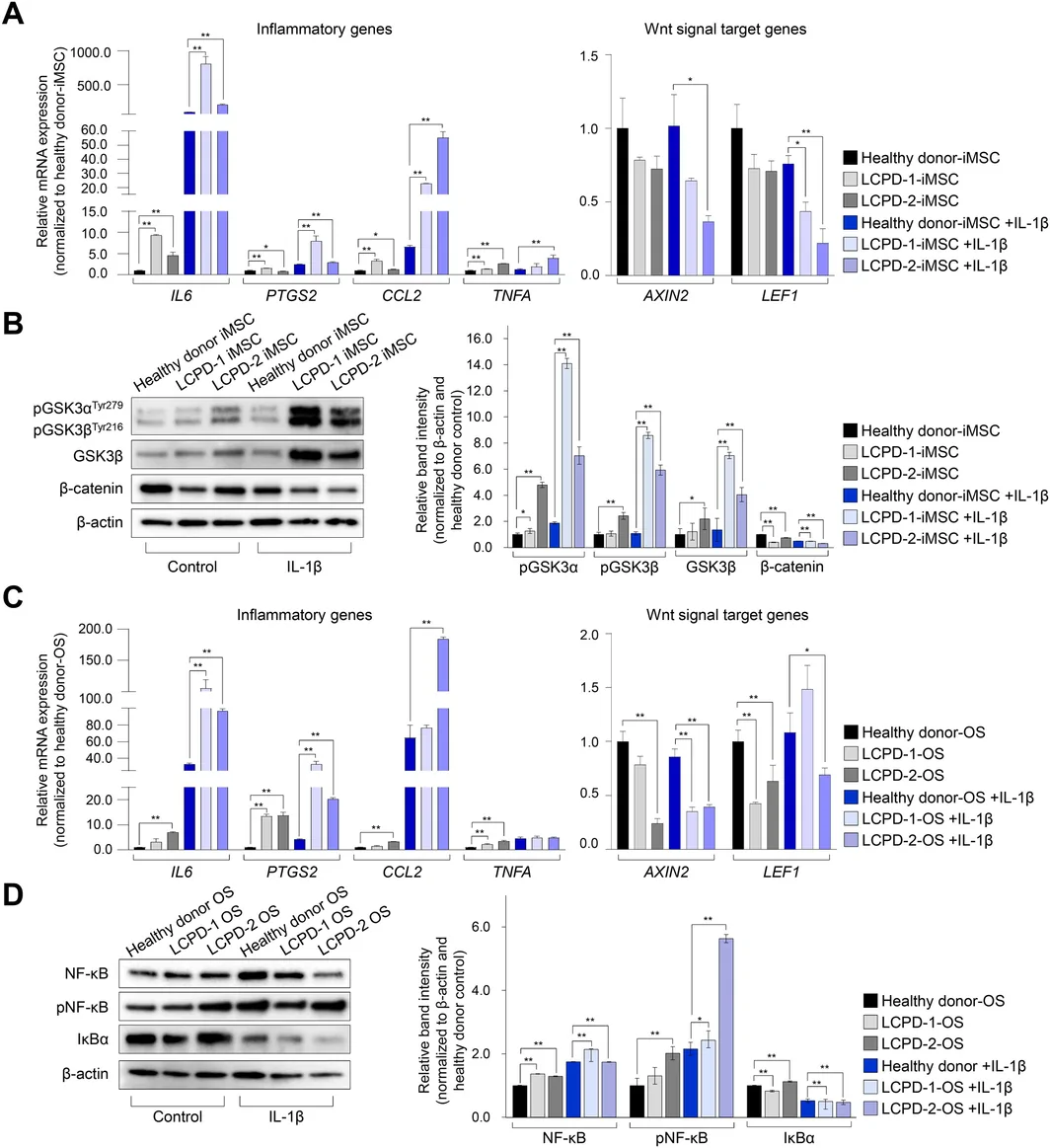
Inflammatory susceptibility and altered Wnt/β‐catenin signalling in LCPD‐iMSCs and LCPD‐iMSC‐derived osteogenic differentiated cells (LCPD‐OSs). (A) Quantitative RT‐PCR analysis of inflammatory genes (*IL6*, *PTGS2*, *CCL2*, *TNFA*) and Wnt target genes (*AXIN2*, *LEF1*) in healthy donor‐ and LCPD‐iMSCs with or without IL‐1β stimulation. Expression levels were normalized to *GAPDH*. *n* = 3 independent experiments; * *p* < 0.05 and **p < 0.01 versus healthy donor‐iMSCs. (B) Western blot analysis of total GSK3β, phosphorylated forms (pGSK3α^Tyr279^, pGSK3β^Tyr216^), and β‐catenin in healthy donor‐, LCPD‐1‐ and LCPD‐2‐iMSCs with or without IL‐1β stimulation. Representative blots (left) and corresponding densitometric quantification (right) are shown. Data represent mean ± SD from three independent experiments. (C) Quantitative RT‐PCR of inflammatory (*IL6*, *PTGS2*, *CCL2*, *TNFA*) and Wnt target genes (*AXIN2*, *LEF1*) in healthy donor‐ and LCPD‐OSs with or without IL‐1β stimulation. Expression levels were normalized to GAPDH. *n* = 3; *p < 0.05 and **p < 0.01 versus healthy donor‐OSs. (D) Western blot analysis of total and phosphorylated NF‐κB^Ser536^ and IκBα in healthy donor‐ and LCPD‐OSs with or without IL‐1β stimulation. Representative blots (left) and densitometric quantification (right) are shown. Data are presented as mean ± SD from three independent experiments.

Because GSK3 hyperactivation and reduced β‐catenin stability were evident in LCPD‐iMSCs (Figure 4), we next examined whether these abnormalities altered their response to inflammatory cues. Under basal conditions, *IL6*, *PTGS2* and *CCL2* transcripts were already elevated in LCPD‐1‐ and LCPD‐2‐iMSCs relative to healthy donor‐iMSCs, suggesting a primed inflammatory state (Figure 5A). Following IL‐1β stimulation, all lines upregulated these cytokines; however, LCPD‐iMSCs consistently exhibited larger fold increases, most notably for *IL6* and *PTGS2*. In contrast, *TNFA* expression remained low across groups and showed only minor induction, indicating selective amplification of specific cytokine programs rather than global hyperactivation.

We then assessed the impact of inflammatory stimulation on Wnt signalling. The β‐catenin target genes *AXIN2* and *LEF1* were slightly reduced in LCPD‐iMSCs under basal conditions and were further suppressed by IL‐1β, with the most pronounced decreases observed in LCPD lines (Figure 5A). These results indicate that inflammatory activation not only amplifies cytokine responses but also exacerbates transcriptional downregulation of canonical Wnt signalling in LCPD‐derived MSCs. To determine whether these transcript‐level changes were associated with altered GSK3 activity, we analysed GSK3α and GSK3β phosphorylation (Figure 5B). At baseline, LCPD‐iMSCs showed higher levels of pGSK3α^Tyr279^ and pGSK3β^Tyr216^ than healthy donor‐iMSCs, consistent with the hyperactive GSK3 state observed in Figure 4. IL‐1β stimulation further elevated phosphorylation of both isoforms in all lines, but LCPD‐iMSCs exhibited greater increases, reinforcing the notion that inflammatory stimuli augment GSK3 activation more strongly in the patient‐derived cells. Total GSK3β remained higher in LCPD‐iMSCs irrespective of stimulation, whereas β‐catenin protein levels were already reduced at baseline and declined even further after IL‐1β, contrasting with only modest reductions in healthy donor‐iMSCs.

To determine whether the exaggerated inflammatory phenotype persists after differentiation, we examined osteogenically differentiated cells (healthy donor‐OS, LCPD‐1‐OS, LCPD‐2‐OS). Consistent with the iMSC data, *IL6*, *PTGS2* and *CCL2* levels were higher in LCPD‐OS cells at baseline and showed greater induction following IL‐1β exposure (Figure 5C). Furthermore, *AXIN2* and *LEF1* remained reduced in LCPD‐OS cells and were further suppressed by IL‐1β, indicating that impaired Wnt signalling is maintained during osteogenic commitment. Western blot analysis corroborated these findings: pNF‐κB levels increased more strongly in LCPD‐OS cells, accompanied by greater IκBα degradation, whereas total NF‐κB p65 protein remained unchanged (Figure 5D). These results demonstrate that LCPD‐iMSCs and their osteogenic derivatives exhibit a persistent pro‐inflammatory bias and increased sensitivity to IL‐1β–induced NF‐κB activation.

Transcriptomic profiling of LCPD fibroblasts and iMSCs revealed molecular alterations associated with inflammatory signalling, extracellular matrix (ECM) organization, and Wnt‐related regulatory pathways (Figures S3 and S4). In LCPD fibroblasts, selected‐gene analysis showed increased expression of inflammatory chemokines and adhesion‐associated genes, including *CXCL2*, *CXCL6*, *CXCL8* and *VCAM1*, together with altered expression of the ECM‐associated regulators *CXCL12* and *TGFB1* (Figure S3C). Increased expression of the Wnt antagonists *SFRP4* and *SOST*, accompanied by reduced expression of *WNT2* and *LEF1*, further suggested dysregulation of Wnt‐related gene expression (Figure S3C and Table S3). Similarly, LCPD‐iMSCs exhibited altered expression of genes associated with ECM remodelling and inflammatory responses, including *CXCL12*, *MMP9*, *PTGS2* and *CCL2* (Figure S4A and Table S4). These changes were accompanied by increased expression of multiple extracellular Wnt antagonists, including *DKK1*, *SFRP1*, *SFRP2*, *WIF1* and *SOST*, together with reduced *CTNNB1* expression (Figure S4A). Although the specific differentially expressed genes differed between fibroblasts and iMSCs, consistent with their distinct cellular identities, GO and KEGG enrichment analyses demonstrated convergent alterations in inflammatory signalling, ECM organization, and developmental signalling pathways in both cell types (Figures S3D and S4B). Collectively, these transcriptomic findings provide molecular context for the enhanced inflammatory responsiveness and altered Wnt/β‐catenin signalling observed in the subsequent functional analyses.

### 3.6 Pharmacological Inhibition of GSK3 Restores Impaired Osteogenic and Adipogenic Differentiation in LCPD‐iMSCs

**FIGURE 6:**
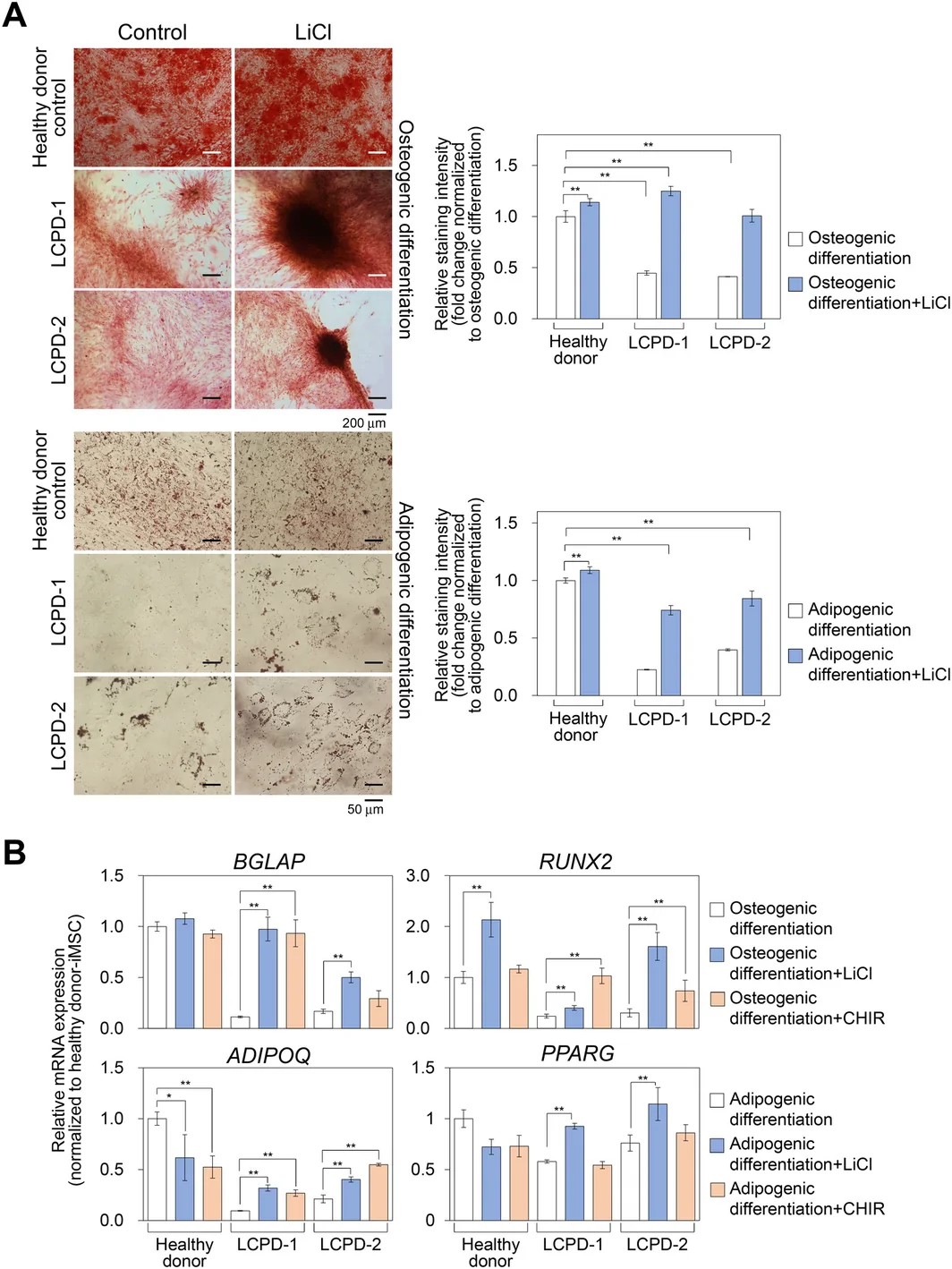
Rescue of osteogenic and adipogenic differentiation defects in LCPD‐iMSCs by GSK3β inhibition. (A) Representative images of Alizarin Red S staining for osteogenesis and Oil Red O staining for adipogenesis in healthy donor‐, LCPD‐1‐ and LCPD‐2‐iMSCs cultured under control or LiCl‐treated conditions. Scale bars = 200 μm (osteogenic) and 50 μm (adipogenic). Relative staining intensity was normalized to the corresponding untreated healthy donor control group, which was set to 1, and is presented as fold change. (B) Quantitative RT‐PCR analysis of osteogenic (*BGLAP*, *RUNX2*) and adipogenic (*ADIPOQ*, *PPARG*) gene expression in iMSCs treated with LiCl or CHIR99021. Data are normalized to GAPDH and presented relative to untreated iMSCs. *n* = 3 independent experiments; *p < 0.05 and **p < 0.01 versus untreated controls.

Given that LCPD‐iMSCs exhibit reduced β‐catenin levels, enhanced GSK3α/β activation, and heightened inflammatory suppression of Wnt signalling, we next investigated whether pharmacological inhibition of GSK3 could functionally restore their impaired mesenchymal differentiation capacity (Figure 6A,B). Because osteogenic and adipogenic differentiation were most severely affected in LCPD‐iMSCs (Figure 3), rescue experiments were focused on these two lineages. During osteogenic induction, untreated LCPD‐1‐ and LCPD‐2‐iMSCs formed markedly fewer mineralized nodules, accompanied by visibly weaker Alizarin Red S staining compared with healthy donor‐iMSCs (Figure 6A). Treatment with LiCl substantially enhanced calcium deposition in both LCPD‐derived lines, resulting in staining patterns that approached those of healthy donor‐iMSCs. Quantitative analysis of Alizarin Red S staining confirmed that LiCl treatment significantly restored mineralization capacity in LCPD‐iMSCs. A similar rescue effect was observed during adipogenic differentiation. Consistent with earlier findings (Figure 3), untreated LCPD‐iMSCs displayed reduced lipid droplet accumulation under adipogenic conditions (Figure 6A). LiCl treatment significantly increased Oil Red O staining intensity in both LCPD‐1‐ and LCPD‐2‐iMSCs, indicating enhanced lipid accumulation relative to untreated patient‐derived cells. Together, these results demonstrate that inhibition of GSK3 effectively improves both osteogenic and adipogenic differentiation outcomes in LCPD‐iMSCs. qRT‐PCR analyses further supported these phenotypic observations. Pharmacological inhibition of GSK3, using either LiCl or the selective inhibitor CHIR99021, significantly increased the expression of key osteogenic markers (*BGLAP*, *RUNX2*) and adipogenic regulators (*ADIPOQ*, *PPARG*) in both LCPD‐derived iMSC lines (Figure 6B). These findings indicate that excessive GSK3 activity contributes directly to blunted lineage‐specific gene induction and that suppression of GSK3 can reactivate differentiation programs that remain intrinsically responsive. Collectively, these data demonstrate that the osteogenic and adipogenic differentiation defects observed in LCPD‐iMSCs arise from a reversible disruption of the GSK3‐β‐catenin signalling axis, rather than irreversible cellular dysfunction. Restoration of β‐catenin signalling output through GSK3 inhibition reinstates lineage‐specific gene expression and functional differentiation. When integrated with the exaggerated inflammatory suppression of Wnt signalling and the transcriptomic alterations associated with extracellular matrix organization, cell adhesion and Wnt‐related pathways (Figures S3 and S4), these results support a model in which LCPD‐derived MSCs exist in a signalling environment characterized by persistently elevated GSK3 activity and insufficient β‐catenin‐mediated transcription. Together, these findings identify GSK3 as a central regulatory node underlying mesenchymal dysfunction in LCPD.

## 4 Discussion

Legg‐Calvé‐Perthes disease (LCPD) is an idiopathic paediatric disorder characterized by avascular necrosis of the femoral head and shares pathological, molecular and clinical features with adult osteonecrosis of the femoral head (ONFH) [3, 4]. Ischemic injury disrupts trabecular bone structure and cartilage integrity, eventually resulting in femoral head deformation and early‐onset osteoarthritis [3, 4]. Preclinical piglet and rodent models have reproduced these pathological changes and demonstrated that ischemia induces robust inflammatory and angiogenic responses [29, 30, 31]. Increased IL‐6, VEGF and HMGB1 levels in patients and ischemic animal models further suggest that inflammation actively contributes to disease progression rather than merely reflecting secondary consequences of disrupted perfusion [31, 32, 33].

Given the central role of mesenchymal stromal/stem cells (MSCs) in bone repair, cartilage maintenance and skeletal growth, MSC dysfunction has long been proposed as a contributor to osteonecrosis. Clinical studies have associated LCPD with adiposity‐related alterations, including elevated circulating leptin levels and increased adipose tissue within the affected femoral head [33]. However, these observations reflect systemic metabolic alterations and tissue‐level remodelling rather than directly demonstrating enhanced intrinsic adipogenic differentiation of mesenchymal progenitor cells. Likewise, damage‐associated molecular patterns released from the necrotic femoral head have been shown to suppress osteogenic differentiation and promote fibrogenic responses in MSCs, highlighting the substantial influence of the local pathological microenvironment on mesenchymal cell behaviour [34].

To distinguish these extrinsic pathological influences from intrinsic cellular properties, we established a patient‐derived iPSC‐MSC model and evaluated mesenchymal differentiation under standardized culture conditions. Under these conditions, LCPD‐iMSCs exhibited impaired osteogenic, adipogenic and chondrogenic differentiation, with osteogenic and adipogenic differentiation being more severely affected than chondrogenesis. The concurrent attenuation of all three mesenchymal lineages argues against a reciprocal osteogenic‐to‐adipogenic lineage shift and instead supports a broader impairment of mesenchymal differentiation competence. Therefore, the reduced adipogenic differentiation observed in our study should not be interpreted as contradicting the clinical pathology of LCPD. Rather, it suggests that the intrinsic defect in LCPD‐derived mesenchymal cells primarily compromises overall differentiation competence, whereas the increased adipose tissue observed in vivo likely reflects the combined effects of intrinsic cellular abnormalities together with ischemia‐driven tissue remodelling, inflammatory signalling, vascular insufficiency, and alterations of the bone marrow microenvironment.

The reproducibility of this phenotype across two independently established LCPD iPSC‐MSC lines further supports that these abnormalities represent a consistent disease‐associated cellular phenotype rather than experimental variation. Accordingly, our iPSC‐MSC platform complements, rather than contradicts, pathological observations obtained from patients and experimental models by isolating the intrinsic mesenchymal component of LCPD. This patient‐specific platform therefore provides a valuable human model for investigating the cellular mechanisms underlying LCPD pathogenesis and their consequences for skeletal regeneration.

A major mechanistic insight emerging from this study is the identification of GSK3α/β hyperactivation and reduced β‐catenin availability as central regulators of lineage impairment in LCPD‐MSCs. Canonical Wnt/β‐catenin signalling drives MSC commitment toward osteoblasts through β‐catenin stabilization and activation of osteogenic transcriptional programs such as RUNX2 and Osterix [17, 18, 19, 20, 35]. Wnt signalling also inhibits adipogenesis by suppressing PPARγ and C/EBPα expression [36, 37, 38] and supports chondrogenic maturation through coordinated matrix regulation. Thus, reduced β‐catenin levels in LCPD‐MSCs are sufficient to explain the observed osteogenic defect and may also contribute to impaired chondrogenesis. However, the concurrent suppression of adipogenesis indicates involvement of a broader signalling imbalance extending beyond classical Wnt pathway regulation. Consistent with this interpretation, LCPD‐iMSCs exhibited elevated phosphorylation of both GSK3α (Tyr279) and GSK3β (Tyr216), increased GSK3B expression, and reduced β‐catenin protein. These findings demonstrate a heightened GSK3 activation state across both isoforms and provide a mechanistic basis for diminished Wnt pathway output. Inflammatory stimulation further amplified this imbalance. IL‐1β exposure increased GSK3α/β phosphorylation and accelerated β‐catenin loss more strongly in LCPD‐iMSCs than in controls, while simultaneously triggering higher induction of IL6, PTGS2, CCL2 and TNFA. This exaggerated inflammatory sensitivity was maintained during osteogenic differentiation, indicating that LCPD‐derived MSCs remain vulnerable to inflammatory cues across developmental states.

Transcriptomic profiling supports these functional observations. Although differential gene expression changes were moderate, LCPD fibroblasts and iMSCs displayed enrichment of pathways associated with extracellular matrix organization, cell adhesion, PI3K‐Akt and MAPK signalling, and NF‐κB regulation. These pathways influence MSC biomechanics, niche interactions, inflammatory responsiveness and lineage competency. Although transcriptomic alterations alone cannot fully account for the functional deficits, their directional alignment with the observed signalling abnormalities suggests that LCPD cells occupy a broader molecular environment prone to β‐catenin instability, GSK3 hyperactivation, and heightened inflammatory reactivity. Importantly, the core defects identified in LCPD‐iMSCs were pharmacologically reversible. GSK3 inhibition with lithium chloride restored β‐catenin levels, enhanced mineralized nodule formation, and increased expression of osteogenic markers, while CHIR99021 produced comparable rescue effects. The simultaneous improvement of adipogenic and chondrogenic markers further indicates that reinstating β‐catenin signalling helps reestablish overall mesenchymal responsiveness. These findings parallel previous observations demonstrating the beneficial impact of Wnt activation on osteogenesis in ONFH models [13, 39] and suggest that LCPD‐associated lineage impairment arises from a reversible signalling imbalance rather than irreversible cellular dysfunction. β‐catenin interacts closely with inflammatory pathways, including NF‐κB and hypoxia‐responsive mediators such as HIF‐1α [12, 31, 40]. Dysregulation of these networks in LCPD may create a reciprocal feedback loop in which inflammatory activation sustains GSK3 activity, further destabilizing β‐catenin and compromising regeneration. This interplay provides a mechanistic framework linking ischemia‐induced inflammation, metabolic stress, and impaired MSC differentiation in LCPD.

In summary, this study identifies hyperactive GSK3 signalling and suppressed β‐catenin activity as key drivers of MSC dysfunction in LCPD. These abnormalities contribute to reduced osteogenic, adipogenic and chondrogenic differentiation capacities and heightened inflammatory sensitivity. Pharmacological inhibition of GSK3 effectively rescues these defects, underscoring the therapeutic potential of targeting the GSK3/β‐catenin axis. Furthermore, the LCPD‐iMSC model offers a robust, patient‐specific platform for mechanistic studies and for developing targeted regenerative interventions for paediatric osteonecrosis and related skeletal disorders.

## Author Contributions

**Binna Seol:** methodology, investigation, writing – original draft, writing – review and editing, formal analysis. **Hyung Jin Lim:** methodology, investigation, writing – review and editing. **Cho Lok Song:** methodology, investigation. **Jungwoon Lee:** methodology, formal analysis, writing – review and editing. **Yee Sook Cho:** conceptualization, methodology, validation, resources, supervision, project administration, funding acquisition, writing – original draft, writing – review and editing.

## Funding

Funded by grants from the Korean Fund for Regenerative Medicine (23A0106L1), the KRIBB research initiative program (KGM1232612) and the National Research Foundation of Korea (NRF) grant (RS‐2023‐00272798).

## Conflicts of Interest

The authors declare no conflicts of interest.

## Supporting information


**Figure S1:** Karyotype analysis of healthy donor‐ and LCPD‐derived iPSCs. Representative G‐banding karyotype images of healthy donor‐, LCPD‐1‐ and LCPD‐2‐iPSCs at passages 15–20. All iPSC lines exhibited a normal chromosomal complement without detectable structural or numerical abnormalities.
**Figure S2:** Growth kinetics of healthy donor‐ and LCPD‐derived iMSCs. Cell proliferation curves of healthy donor‐, LCPD‐1‐ and LCPD‐2‐iMSCs measured over 6 days at passages 9–11. Viable cell numbers were counted daily, and all iMSC lines showed comparable proliferation rates under standard culture conditions. Data are presented as the mean ± SD from three independent experiments.
**Figure S3:** Comparative transcriptomic profiling of LCPD patient fibroblasts. A. Hierarchical clustering heatmap showing differentially expressed genes (DEGs) in LCPD‐1 and LCPD‐2 fibroblasts relative to healthy donor fibroblasts. B. Venn diagram of differentially expressed genes (DEGs) identified in LCPD‐1 versus healthy donor and LCPD‐2 versus healthy donor fibroblasts using thresholds of fold change > 2 and adjusted *p* < 0.05. Totals and sector labels indicate upregulated (red), downregulated (blue), and contra‐regulated (opposite‐direction) genes. Across both patient comparisons, 1710 DEGs were detected, including 800 shared upregulated, 879 shared downregulated, and 31 contra‐regulated genes. C. Heatmap showing log_2_ fold changes of selected genes associated with inflammatory/adhesion signalling, ECM/developmental signalling, and Wnt pathway regulation in LCPD‐1 and LCPD‐2 fibroblasts relative to healthy donor fibroblasts. D. Functional enrichment of DEGs. Bar plots show the top Gene Ontology (GO, Biological Process) terms and Kyoto Encyclopedia of Genes and Genomes (KEGG) pathways enriched among up‐ and downregulated genes. Bars indicate gene counts; overlaid lines denote −log_10_(p) values.
**Figure S4:** Transcriptomic enrichment analysis of DEGs in LCPD‐iMSCs. A. Heatmap showing log_2_‐normalized read counts of selected genes associated with ECM/inflammatory signalling and Wnt pathway regulation in healthy donor‐iMSC and LCPD‐2‐iMSC. Log_2_ fold changes (LCPD‐2‐iMSC relative to healthy donor‐iMSC) are indicated alongside the heatmap. B. GO and KEGG pathway enrichment analyses of significantly upregulated and downregulated genes in LCPD‐iMSCs compared with healthy donor‐iMSCs. The bar plots display the top 10 enriched GO biological processes (left) and KEGG pathways (right). Bars represent gene counts, and the overlaid line indicates −log_10_(p) values.
**Table S1:** List of primary and secondary antibodies used.
**Table S2:** Primers used for quantitative RT‐PCR.
**Table S3:** Log2 fold change values of selected genes from mRNA‐seq analysis in LCPD fibroblasts relative to healthy donor fibroblasts.
**Table S4:** Log2 fold change values of selected genes from mRNA‐seq analysis in LCPD‐2‐iMSCs relative to healthy donor iMSCs.

## Data Availability

The RNA‐sequencing data generated in this study have been deposited in the NCBI Gene Expression Omnibus (GEO) under accession number GSE342619. The dataset is currently private during peer review and will be made publicly available in accordance with GEO release requirements.
