# Supplementary material for: Patient‐Derived iPSC‐MSC Modelling Reveals Reversible GSK3‐Mediated Suppression of Mesenchymal Lineage Differentiation in Legg‐Calvé‐Perthes Disease
Source: J Cell Mol Med. 2026 Sep 7;30(17):e71350. doi: 10.1111/jcmm.71350 (PMC13550505; doi:10.1111/jcmm.71350)
Supplement: Supplementary file 1 [file JCMM-30-e71350-s001.docx]

**Supplemental Information**

**Supplementary Figure S1.** Karyotype analysis of healthy donor- and LCPD-derived iPSCs. Representative G-banding karyotype images of healthy donor-, LCPD-1-, and LCPD-2-iPSCs at passages 15-20. All iPSC lines exhibited a normal chromosomal complement without detectable structural or numerical abnormalities.

**Supplementary Figure S2.** Growth kinetics of healthy donor- and LCPD-derived iMSCs. Cell proliferation curves of healthy donor-, LCPD-1-, and LCPD-2-iMSCs measured over six days at passages 9-11. Viable cell numbers were counted daily, and all iMSC lines showed comparable proliferation rates under standard culture conditions. Data are presented as the mean ± SD from three independent experiments.

**Supplementary Figure S3.** Comparative transcriptomic profiling of LCPD patient fibroblasts. A. Hierarchical clustering heatmap showing differentially expressed genes (DEGs) in LCPD-1 and LCPD-2 fibroblasts relative to healthy donor fibroblasts. B. Venn diagram of differentially expressed genes (DEGs) identified in LCPD-1 vs. healthy donor and LCPD-2 vs. healthy donor fibroblasts using thresholds of fold change > 2 and adjusted p < 0.05. Totals and sector labels indicate upregulated (red), downregulated (blue), and contra-regulated (opposite-direction) genes. Across both patient comparisons, 1,710 DEGs were detected, including 800 shared upregulated, 879 shared downregulated, and 31 contra-regulated genes. C. Heatmap showing log_2_ fold changes of selected genes associated with inflammatory/adhesion signaling, ECM/developmental signaling, and Wnt pathway regulation in LCPD-1 and LCPD-2 fibroblasts relative to healthy donor fibroblasts. D. Functional enrichment of DEGs. Bar plots show the top Gene Ontology (GO, Biological Process) terms and Kyoto Encyclopedia of Genes and Genomes (KEGG) pathways enriched among up- and downregulated genes. Bars indicate gene counts; overlaid lines denote -log10(p) values.

**Supplementary Figure S4.** Transcriptomic enrichment analysis of DEGs in LCPD-iMSCs. A. Heatmap showing log_2_-normalized read counts of selected genes associated with ECM/inflammatory signaling and Wnt pathway regulation in healthy donor-iMSC and LCPD-2-iMSC. Log_2_ fold changes (LCPD-2-iMSC relative to healthy donor-iMSC) are indicated alongside the heatmap. B. GO and KEGG pathway enrichment analyses of significantly upregulated and downregulated genes in LCPD-iMSCs compared with healthy donor-iMSCs. The bar plots display the top 10 enriched GO biological processes (left) and KEGG pathways (right). Bars represent gene counts, and the overlaid line indicates -log10(p) values.

**Supplementary Table S1.** List of primary and secondary antibodies used.

| **Antibodies** | **Target protein** | **Company (Cat. No.)** | **Dilution** |
| --- | --- | --- | --- |
| Primary antibodies | OCT4 | Santa Cruz (sc-9081) | 1 : 100 |
|  | NANOG | R&D (AF1997) | 1 : 40 |
|  | SSEA-3 | R&D (MAB1434) | 1 : 30 |
|  | SSEA-4 | Millipore (MAB1435) | 1 : 30 |
|  | TRA-1-60 | Millipore (MAB4360) | 1 : 100 |
|  | TRA-1-81 | Millipore (MAB4381) | 1 : 100 |
|  | TUJ1 | Biolegend (802001) | 1 : 500 |
|  | NESTIN | Millipore (MAB5326) | 1 : 100 |
|  | α-SMA | Sigma (A5228) | 1 : 200 |
|  | DESMIN | Chemicon (AB907) | 1 : 50 |
|  | SOX17 | R&D (MAB1924) | 1 : 50 |
|  | FOXA2 | Millipore (07-633) | 1 : 100 |
|  | β-catenin | Abcam (ab22656) | 1 : 1000 |
|  | CD73-PE | Miltenyl Biotec (130-095-182) | 1 : 11 |
|  | CD105-PE | Miltenyl Biotec (130-112-321） | 1 : 11 |
|  | CD90-PE | Miltenyl Biotec (130-114-860) | 1 : 11 |
|  | CD34/CD45 Cocktail, FITC, PE | Thermo Fisher (MA1-12221) | 1 : 11 |
|  | FITC anti-HLA-A,B,C | BioLegend (311404) | 1 : 11 |
|  | FITC anti-HLA-DR | BioLegend (307604) | 1 : 11 |
|  | FITC Mouse IgG2a, Isotype Ctrl | BioLegend (400210) | 1 : 200 |
|  | PE Mouse IgG1 isotype control | Miltenyl Biotec (130-113-200) | 1 : 50 |
|  | GSK3 β | Abcam (ab32391) | 1:5000 |
|  | p-GSK3 α/β | Santa Cruz (sc-81496) | 1:200 |
|  | NF-κB p65 | Cell Signaling Technology (8242S) | 1:1000 |
|  | Phospho-NF-κB p65 | Cell Signaling Technology (3033S) | 1:1000 |
|  | IκBα | Cell Signaling Technology (4812S) | 1:1000 |
| Secondary antibodies | Chicken anti-rabbit IgG | Invitrogen (A21442) | 1 : 200 |
|  | Chicken anti-goat IgG | Invitrogen (A21467) | 1 : 200 |
|  | Goat anti-mouse IgM | Invitrogen (A21044) | 1 : 200 |
|  | FITC mouse anti-rat IgM | BD Pharmingen (553887) | 1 : 200 |
|  | Donkey anti-mouse IgG | Invitrogen (A21202) | 1 : 200 |
|  | FITC rat anti-mouse IgM | BD Pharmingen (553437) | 1 : 200 |
|  | Goat anti-rabbit IgG-HRP | Santa Cruz (SC-2004) | 1 : 10000 |
|  | Goat anti-mouse IgG -HRP | Ab Frontier (LF-SA8001) | 1 : 10000 |

**Supplementary Table S2.** Primers used for quantitative RT-PCR.

|  | **Gene** | **Sequence (5' → 3')** | |
| --- | --- | --- | --- |
| Pluripotency | *OCT4* | *Forward* | *GAG AAG GAT GTG GTC CGA GTG TG* |
|  |  | *Reverse* | *CAG AGG AAA GGA CAC TGG TCC C* |
|  | *REX1* | *Forward* | *AAT GCG TCA TAA GGG GTG AG* |
|  |  | *Reverse* | *TCA ATG CCA GGT ATT CCT CC* |
| Ectoderm | *SOX1* | *Forward* | *GCT GAC ACC AGA CTT GGG TT* |
|  |  | *Reverse* | *CCT CTC GCC TCG TTT TGA CT* |
|  | *PAX6* | *Forward* | *GCC AGC AAC ACA CCT AGT CA* |
|  |  | *Reverse* | *TGT GAG GGC TGT GTC TGT TC* |
| Mesoderm | *HAND1* | *Forward* | *TTC ATC CTG CCA TCC CCA AC* |
|  |  | *Reverse* | *GCA TAG ACA CTG CTT TGG CG* |
|  | *PDGFRA* | *Forward* | *TGT GGG ACA TTC ATT GCG GA* |
|  |  | *Reverse* | *ACT GCC AGC TCA CTT CAC TC* |
| Endoderm | *SOX7* | *Forward* | *ACG GCC ACG TAC TAC AAC AG* |
|  |  | *Reverse* | *AAC GTG GGG AAG GGG ATA GA* |
|  | *AFP* | *Forward* | *ACT GCA ATT GAG AAA CCC ACT GGA GAT G* |
|  |  | *Reverse* | *CGA TGC TGG AGT GGG CTT TTT GTG T* |
| Osteogenesis | *BGLAP* | *Forward* | *CAA AGG TGC AGC CTT TGT GTC* |
|  |  | *Reverse* | *TCA CAG TCC GGA TTG AGC TCA* |
|  | *RUNX2* | *Forward* | *CGC CTC ACA AAC AAC CAC AG* |
|  |  | *Reverse* | *TCA CTG TGC TGA AGA GGC TG* |
| Adipogenesis | *ADIPOQ* | *Forward* | *AAA GGA GAT CCA GGT CTT ATT GGT* |
|  |  | *Reverse* | *GTT CTC CTT TCC TGC CTT GGA* |
|  | *PPARG* | *Forward* | *TGG AAT TAG ATG ACA GCG ACT TGG* |
|  |  | *Reverse* | *CTG GAG CAG CTT GGC AAA CA* |
| Chondrogenesis | *SOX9* | *Forward* | *AGC GCC CCC ACT TTT GCT CT* |
|  |  | *Reverse* | *GCT CGC CCT TGG GGA ACG TG* |
|  | *COL2A1* | *Forward* | *CTG GTG ATG ATG GTG AAG* |
|  |  | *Reverse* | *CCT GGA TAA CCT CTG TGA* |
| Wnt/β-catenin signaling | *CTNNB1* | *Forward* | *CCTGCCATCTGTGCTCTTCG* |
|  |  | *Reverse* | *CGC TGG GTA TCC TGA TGT GC* |
|  | *GSK3B* | *Forward* | *CAC ACC TGC ACT CTT CAA CTT CAC* |
|  |  | *Reverse* | *TCT GTC CAC GGT CTC CAG TAT TAG* |
| Inflammatory genes | *IL6* | *Forward* | *CTT CGG TCC AGT TGC CTT CT* |
|  |  | *Reverse* | *TGG AAT CTT CTC CTG GGG GT* |
|  | *PTGS2* | *Forward* | *GTT CCA CCC GCA GTA CAG AA* |
|  |  | *Reverse* | *AGG GCT TCA GCA TAA AGC GT* |
|  | *CCL2* | *Forward* | *CCT TCA TTC CCC AAG GGC TC* |
|  |  | *Reverse* | *GGT TTG CTT GTC CAGG TGG T* |
|  | *TNF* | *Forward* | *CAA GGA CAG CAG AGG ACC AG* |
|  |  | *Reverse* | *TCC TTT CCA GGG GAG AGA GG* |
| Wnt signal target genes | *AXIN2* | *Forward* | *TAA CCC CTC AGA GCG ATG GA* |
|  |  | *Reverse* | *AGT TCC TCT CAG CAA TCG GC* |
|  | *LEF1* | *Forward* | *TGC ATC AGG TAC AGG TCC AAG* |
|  |  | *Reverse* | *ACG TTG GGA ATG AGC TTC GT* |
| Housekeeping | *GAPDH* | *Forward* | *GAA GGT GAA GGT CGG AGT C* |
|  |  | *Reverse* | *GAA GAT GGT GAT GGG ATT TC* |

**Supplementary Table S3.** Log_2_ fold change values of selected genes from mRNA-seq analysis in LCPD fibroblasts relative to healthy donor fibroblasts.

| **Group** | **Gene symbol** | **LCPD-1 / Healthy donor** | **LCPD-2 / Healthy donor** | **LCPD-1** | **LCPD-2** |
| --- | --- | --- | --- | --- | --- |
|  |  | **Fold change** | | **Log_2_FC** | |
| Inflammation / NF-κB | *CXCL2* | 6.72 | 16.50 | 2.75 | 4.05 |
|  | *CXCL6* | 5.86 | 10.57 | 2.55 | 3.40 |
|  | *CXCL8* | 2.53 | 6.45 | 1.34 | 2.69 |
|  | *VCAM1* | 6.21 | 3.05 | 2.63 | 1.61 |
| ECM / developmental signaling | *CXCL12* | 2.17 | 3.93 | 1.12 | 1.97 |
|  | *TGFB1* | 2.80 | 2.94 | 1.49 | 1.56 |
| Wnt inhibition | *SFRP4* | 3.71 | 7.21 | 1.89 | 2.85 |
|  | *SOST* | 201.72 | 28.44 | 7.66 | 4.83 |
| Wnt signaling | *WNT2* | 0.07 | 0.05 | -3.83 | -4.43 |
|  | *LEF1* | 0.23 | 0.25 | -2.15 | -2.00 |

**Supplementary Table S4.** Log_2_ fold change values of selected genes from mRNA-seq analysis in LCPD-2-iMSCs relative to healthy donor iMSCs.

| **Group** | **Gene symbol** | **LCPD-2/ Healthy donor** | **LCPD-2** |
| --- | --- | --- | --- |
|  |  | **Fold change** | **Log_2_FC** |
| ECM / niche remodeling | *CXCL12* | 131.13 | 7.04 |
|  | *MMP9* | 104.67 | 6.71 |
| Inflammation / NF-κB | *PTGS2* | 4.70 | 2.23 |
|  | *CCL2* | 16.98 | 4.09 |
| Wnt antagonists | *DKK1* | 2.02 | 1.02 |
|  | *SFRP1* | 61.36 | 5.94 |
|  | *SFRP2* | 1170.87 | 10.19 |
|  | *WIF1* | 2.08 | 1.06 |
|  | *SOST* | 2.13 | 1.09 |
| Wnt core signaling | *CTNNB1* | 0.52 | −0.941 |
